# Extensive Alternative Splicing Patterns in Systemic Lupus Erythematosus Highlight Sexual Differences

**DOI:** 10.3390/cells12232678

**Published:** 2023-11-22

**Authors:** Despoina Kosmara, Sofia Papanikolaou, Christoforos Nikolaou, George Bertsias

**Affiliations:** 1 Rheumatology and Clinical Immunology, University Hospital of Heraklion and University of Crete Medical School, 71500 Heraklion, Greece; 2 Foundation for Research and Technology-Hellas (FORTH), Infections and Immunity, Institute of Molecular Biology and Biotechnology, 71110 Heraklion, Greece; 3 Biomedical Sciences Research Center “Alexander Fleming”, Institute of Bioinnovation, 16672 Athens, Greece

**Keywords:** genomic variation, sex, alternative splicing, autoimmunity, intron retention

## Abstract

Substantial evidence highlights divergences in immune responses between men and women. Women are more susceptible to autoimmunity, whereas men suffer from the more severe presentation of autoimmune disorders. The molecular mechanism of this sexual dimorphism remains elusive. Herein, we conducted a comprehensive analysis of sex differences in whole-blood gene expression focusing on alternative splicing (AS) events in systemic lupus erythematosus (SLE), which is a prototype sex-biased disease. This study included 79 SLE patients with active disease and 58 matched healthy controls who underwent whole-blood RNA sequencing. Sex differences in splicing events were widespread, existent in both SLE and a healthy state. However, we observed distinct gene sets and molecular pathways targeted by sex-dependent AS in SLE patients as compared to healthy subjects, as well as a notable sex dissimilarity in intron retention events. Sexually differential spliced genes specific to SLE patients were enriched for dynamic cellular processes including chromatin remodeling, stress and inflammatory responses. Remarkably, the extent of sexual differences in AS in the SLE patients and healthy individuals exceeded those in gene expression. Overall, this study reveals an unprecedent variation in sex-dependent splicing events in SLE and the healthy state, with potential implications for understanding the molecular basis of sexual dimorphism in autoimmunity.

## 1. Introduction

Autoimmunity includes a wide range of disorders caused by immune responses directed against self-antigens, therefore leading to perpetuated tissue inflammation [1]. A striking characteristic of autoimmune diseases is that they demonstrate a greater frequency in females than in males, yet the latter tend to suffer from more severe autoimmunity presentations [2]. Understanding the molecular basis of sex differences in the context of autoimmune disorders is of considerable importance for diagnostic and therapeutic purposes.

Systemic lupus erythematosus (SLE) is a chronic autoimmune disorder characterized by the production of antinuclear autoantibodies in combination with multisystem inflammation [3]. Lupus has a high female-to-male incidence ratio (up to 12:1) depending on age and ethnicity; however, male SLE patients display an increased frequency of pathogenic anti-DNA autoantibodies and major organ involvement (i.e., from the kidneys) [4,5]. The molecular basis underlying these sex differences remains ill-defined, although it can be explained to some extent by hormonal factors and the incomplete inactivation of X-linked immune-related genes, thus leading to increased expression levels in females [6].

Pre-mRNA splicing is a post-transcriptional process that involves the removal of introns and the connection of exons for creating mature mRNA molecules. This process can result in the inclusion or exclusion of various coding regions in the final transcript, a process known as alternative splicing (AS), which produces unique mRNA isoforms [7]. AS is encountered in approximately 95% of all protein-coding genes containing multiple exons, and it appears in nearly all human organs [8]. It has been suggested that AS may represent a crucial mechanism for gene expression regulation and protein diversity generation, also contributing to the fine-tuning of functional immune responses [9]. Specifically, alternatively spliced isoforms have been detected in many immune-cell-derived genes, including cell surface receptors, cytokines and cytokine receptors, as well as complement proteins and receptors [10,11,12,13]. In the context of autoimmunity, there is circumstantial evidence to implicate a number of alternatively spliced genes such as the interferon regulatory factor 5 (*IRF5*) and B-cell scaffold protein with ankyrin repeats 1 (*BANK1*) in SLE; CD44, TNF-receptor 2 (*TNFR2*) and IL-6 receptor (*IL6R*) in rheumatoid arthritis; IL-7 receptor (*IL7R*) in multiple sclerosis; and C-X-C Motif Chemokine Receptor 3 (*CXCR3*) in inflammatory bowel disease [14,15,16,17]. 

We recently demonstrated splicing to be significantly altered in the whole blood of SLE patients as compared to healthy individuals, with differentially spliced genes showing enrichment for the immune system and type I interferon signaling pathways [18]. In addition, we and others have characterized genetic variants that regulate the splicing behavior (so-called splicing quantitative trait loci) of genes connected to lupus, including *IRF7*, *IRF5*, *TCF7* and *WDFY4* [18,19]. Further analysis revealed that splicing perturbation in SLE is driven primarily by abnormalities in intron retention, which is a process where introns are retained in the final transcript [20,21]. Collectively, these findings highlight an important, previously overlooked layer of molecular variation associated with SLE disease. Although the precise functional relevance of splicing abnormalities is yet to be established, it is notable that intron retention has been proposed as a mechanism for genome regulation and the generation of neoantigens in various malignancies [22,23,24]. 

Immune cells have been shown to display broad sex-biased transcriptional differences both in the unstimulated state and following a challenge with exogenous pathogens or immunization [25,26,27,28,29]. Previously, we identified a blood gene signature that differentiates males versus females with SLE as compared to their healthy counterparts [18]. To this end, only a few studies have explored sex-dependent alternative splicing events at the genome-wide level in unaffected human tissues [30,31,32], yet none have been conducted in the context of autoimmune inflammatory diseases. 

In this work, we present a comprehensive analysis of sex-associated differential splicing events in a dataset of whole-blood samples derived from 79 active SLE patients and 58 healthy individuals. We found that biological sex exerts a considerable impact on splicing dynamics both in the healthy state and in SLE disease. Importantly, we identified sexually differential AS-affected genes in SLE to be enriched in distinct functions associated with chromatin remodeling and cellular stress processes. We also observed increased sexually differentially spliced genes with increased intron retention events, which were mainly confined to male SLE patients and negatively correlated with mRNA expression levels. Overall, we provide a first-level characterization of sex-biased AS events in SLE, which could help to gain novel insights into the molecular correlates of sex differences in autoimmunity and facilitate the development of novel biomarkers and targeted diagnostic and therapeutic approaches.

## 2. Materials and Methods

### 2.1. RNA Sequencing Study in SLE Patients and Healthy Individuals

We analyzed the whole-blood transcriptional profiles of 79 patients with SLE (69 females, 10 males) and 58 age-matched healthy individuals (48 females, 10 males). RNA sequencing and initial data generation were performed as described in our previous study [18]. Briefly, consecutively registered patients with SLE were enrolled from the rheumatology clinics of the participating centers (University Hospital of Heraklion, University Hospital C.F.R Cluj Napoca, General Hospital of Athens ‘Laikon’, ‘Attikon’ University Hospital in Athens and ‘Hippokration’ Hospital of Thessaloniki). Healthy individuals were recruited from the blood transfusion units of the corresponding hospitals. All patients had active disease at the time of blood sampling, defined as a clinical SLE Disease Activity Index (SLEDAI)-2000 [33] (i.e., excluding the serological markers anti-dsDNA and complement levels) greater than 4 [34]. Supplementary Table S1 summarizes the demographic and clinical characteristics of the patients and healthy individuals.

### 2.2. Analysis of Differential Alternative Splicing (AS) Events

AS events were identified utilizing the Vertebrate Alternative Splicing and Transcription Tools (Vast-tools v2.5.1) [35,36] and in accordance with our previously reported pipeline [21]. Briefly, this software aligns the reference genome (hg19) to obtain unmapped reads, subsequently aligned to predefined splice junction libraries. The alignment of the input files was conducted with the Bowtie package (v1.0.0). For the purpose of our study, we categorized the samples into four main groups: healthy female, healthy male, SLE female, and SLE male. Because the biological replicates within each group exhibited a read coverage of approximately 10 million reads per sample, we randomly combined these samples into subgroups, each containing approximately 10 samples, thus helping to balance the read coverage across the main groups (Table 1).

Next, the output files were consolidated into a table that included details about each predefined AS event. This information encompassed the gene symbol, event ID (linked to the VastDB atlas; http://vastdb.crg.eu/; accessed on 23 June 20), and the coordinates and length of the region involved in the AS event, as well as the complete set of coordinates, event type, percent spliced-in, and quality scores [35,36]. Bayesian inference was employed to analyze the differential usage of AS events across the four main groups [37]. The AS events were quantified using the percent spliced-in index (PSI), which represents the percentage of reads supporting the inclusion of the examined AS event in a transcript. Vast-tools statistically evaluates the PSI in a way that may only slightly be affected by the alignment error rates. A splicing event was deemed statistically significant if the difference in the PSI (delta-PSI) between two compared groups of samples was ≥15%. Based on our previous experience [21], we chose this threshold to increase the validity and relevance of the reported AS events. Since functional and genetic association analyses can only be performed at the level of genes, we then mapped our list of defined, significant AS events to the corresponding gene loci in which they resided and used these genes for all our downstream analyses. This inherently suggests that more than one AS event could be associated with a single gene. It should be noted, however, that the number of AS events per gene is more likely correlated with the length of the gene and the complexity of its transcripts (for example, the number of exons) and less with an assumed propensity to be alternatively spliced. We therefore treated every gene in the same way, regardless of the number of AS events it was found to be associated with. Using group-level (Table 1) estimates of disease activity (average SLEDAI-2000), active renal disease (defined as proteinuria ≥ 500 mg/g, with/without active urine sediment) and serological activity (hypocomplementemia and/or increased anti-dsDNA titers), we performed a Spearman’s rank correlation analysis with the PSI values from all the transcripts.

### 2.3. Differential Gene Expression and Pathway Analysis

Differential gene expression analysis was performed on the gene counts using the DESeq2 tool (v1.40.2) [38], setting a 5% *p*-value cutoff and an absolute log2-fold change in order to define statistical significance in the differentially expressed genes (DEGs). The clusterProfiler (v4.8.2) [39] and Metascape (v3.5.20230501) [40] tools were employed for a functional enrichment analysis of the differentially AS genes and DEGs.

## 3. Results

### 3.1. Sex Exerts Widespread Effects on RNA Splicing in SLE and the Healthy State with Enrichment for Intron Retention and Exon Skipping Events

**Figure 1:**
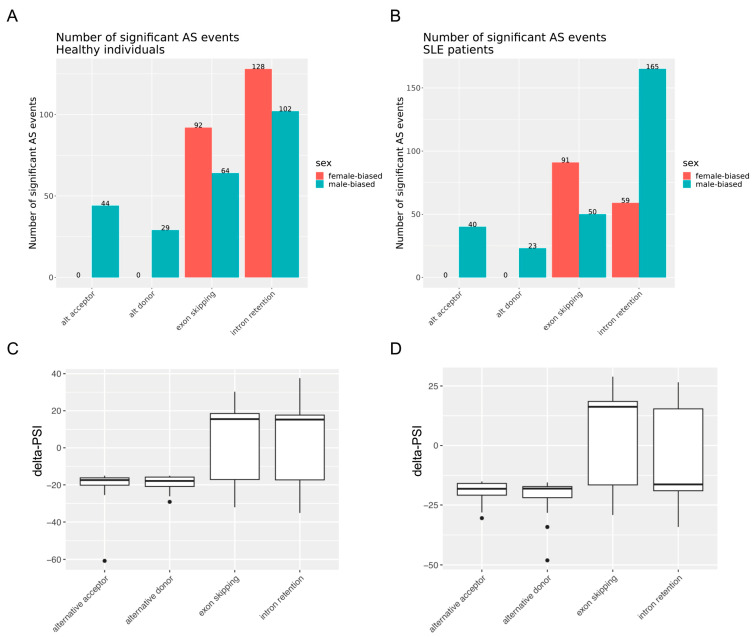
(**A**,**B**) Female-biased and male-biased alternative splicing events grouped into four major classes (alternative acceptor, alternative donor, exon skipping, intron retention) in healthy individuals (**A**) and SLE patients (**B**). The number on top of the bars indicates the actual number of significant alternative splicing events in each group. (**C**,**D**) Box plot distribution of female-minus-male delta-PSI values of significant alternative splicing events across the four classes of events in healthy individuals (**C**) and SLE patients (**D**).

In agreement with our previous study [21], we observed extensive splicing perturbation in the peripheral blood of active SLE patients, with several AS events correlating with overall disease activity (SLEDAI-2000) or activity from the kidney and serological domain (Supplementary Tables S2–S4). Female–male differences in AS in the blood of both SLE patients and healthy individuals were identified. Specifically, we captured a total of 428 and 459 significant sex-dependent AS events in patients with SLE and healthy subjects, respectively (Table 2). We further analyzed these differential splicing events according to sex by focusing on four major classes of discrete AS events, namely, (1) alternative acceptor, where a 3′ splice junction is used to change the 5′ boundary of the downstream exon; (2) alternative donor, where a 5′ junction is utilized to change the 3′ boundary of the upstream exon; (3) exon skipping; and (4) intron retention. Figure 1 illustrates the number of female-biased and male-biased AS events (delta-PSI ≥ 15% in the female-minus-male and male-minus-female comparisons, respectively), stratified by the class of splicing event in the healthy (Figure 1A) and SLE (Figure 1B) states, respectively. Notably, alternative acceptor and donor splicing events occurred exclusively in males (both SLE and healthy).

Intron retention represented the most prevalent class of AS among both SLE patients and healthy individuals (52.3% and 50.1% of sex-biased AS events, respectively) (Figure 1A,B). This predominance of intron retention events comes as a corroboration of recent data reported by us [21] and others [20] in the context of SLE. We observed a remarkable inverse relationship of intron retention events across the sex and disease state subgroups. Specifically, while intron retention events were more frequent in healthy women than in men (128 versus 102), the opposite trend pertained to SLE patients (59 versus 165; *p*-value < 0.00001 according to Fisher’s exact test). By contrast, exon skipping showed a female predominance in both states, albeit with an enhanced female/male ratio in the SLE group (*p* = 0.3412). The aforementioned sex differences are reflected in the distribution of delta-PSI values (female-minus-male) across the four classes of AS event types in the SLE and healthy subjects (Figure 1C,D). Collectively, sex may exert a strong impact on RNA splicing in whole-blood immune cells, and intron retention events are significantly decreased in women compared to men with SLE. 

### 3.2. Alternative Splicing Events Implicate Distinct Molecular Pathways in SLE Disease and the Healthy State

**Figure 2:**
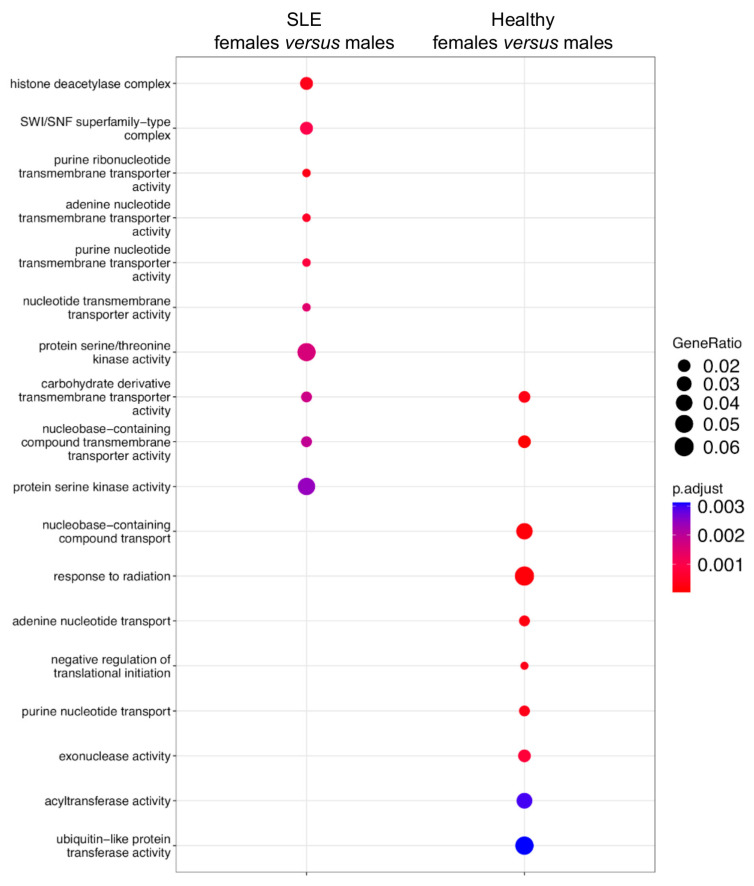
Pathway enrichment analysis of sexually differential spliced genes in SLE and healthy state. Left column contains the pathways enriched in sex-biased alternatively spliced genes in the whole blood of patients with active SLE. Right column contains the corresponding pathways in healthy subjects.

We sought to explore the genes harboring the underlying sex-related AS events. The 428 sexually differential splicing events in the SLE patients were assigned to 398 genes (Supplementary Table S5), whereas the respective 459 AS events in the healthy subjects corresponded to 421 genes (Supplementary Table S6) (Table 2). Of note, only 56 genes were sexually differentially spliced both in SLE and in the healthy state (Supplementary Table S7). To gain a broader understanding of the biological processes targeted by AS, a pathway enrichment analysis was performed for the genes affected by sex-dependent AS. In agreement with the small number of shared genes, we found distinct molecular pathways in SLE versus healthy individuals. Specifically, genes with sex-biased AS in the SLE patients showed enrichment, especially in histone deacetylation and the SWI/SNF complex, which are both linked to chromatin remodeling, followed by the protein serine/threonine kinase activity pathway. In the healthy counterparts, there was enrichment for the nucleobase-containing compound transport and acyltransferase pathways involved in cellular energy, followed by response to radiation and ubiquitin-like protein transferase activity (Figure 2).

**Figure 3:**
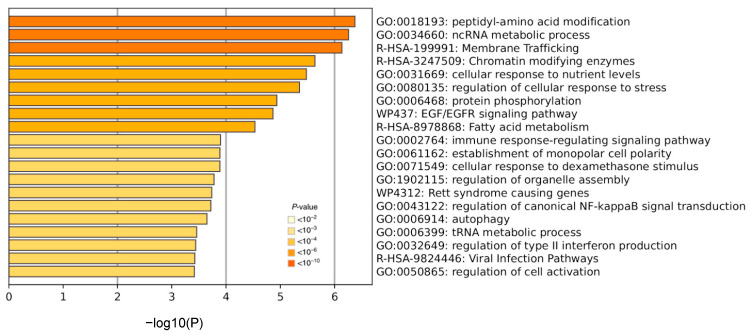
Pathway analysis in genes affected by sex-biased alternative splicing events specifically in the whole blood of SLE patients. The darkness of the orange color reflects the *p*-value of the given term. The darker the color, the more significant the *p*-value is.

Next, we focused on the 342 genes with significant sex-biased AS events in SLE but not in healthy individuals (Supplementary Table S8). In line with the aforementioned data, our pathway analysis showed enrichment for chromatin-modifying enzymes, the cellular response to nutrient levels and stress, EGF/EGFR signaling, the immune response-regulating signaling pathway, the regulation of NF-κB signal transduction and the regulation of type I (viral infection) and type II interferon (Figure 3).

**Figure 4:**
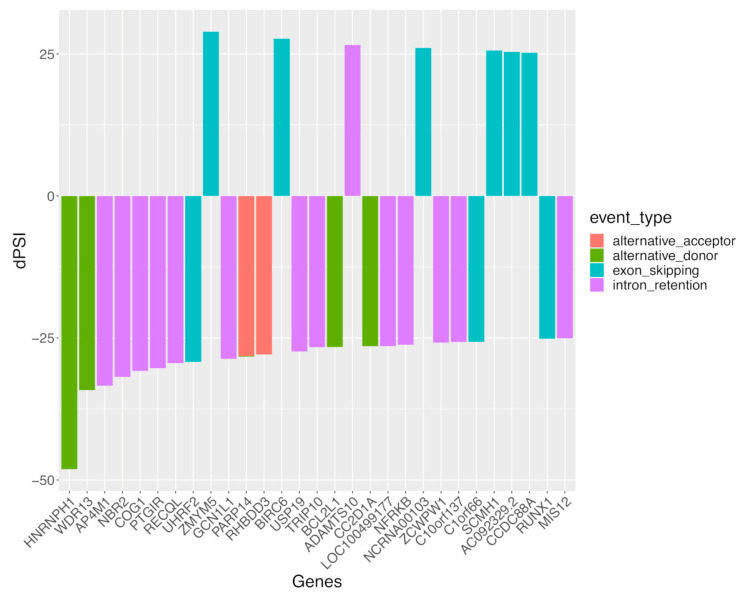
The 30 top-prioritized sexually spliced genes (according to delta-PSI value in the female-minus-male comparison) in the whole blood of patients with active SLE. Colors correspond to different classes of AS events. Positive delta-PSI values indicate female-biased whereas negative values indicate male-biased splicing events.

To gain further insight, we prioritized the top 30 genes with sexual differential AS (DAS), specifically in SLE, according to the female-minus-male delta-PSI value of the underlying AS events (Figure 4). Although the greatest sex difference was noted for the alternative donor splicing event affecting *HNRNPH1* (encoding for heterogeneous nuclear ribonucleoprotein H1), intron retention and exon skipping splicing events were most prevalent, which accords with our result in Figure 1. Of interest, most genes displayed male-biased (i.e., negative delta-PSI values) splicing events. Moreover, some of these genes, such as *USP19* [41] and *RUNX1* [42,43], have been implicated in autoimmunity, therefore providing a rationale for examining how their splice isoforms might regulate immune responses. 

### 3.3. Sex Differences in Alternative Splicing Events Are More Extensive and May Underly Distinct Biological Processes Other Than Differential Gene Expression in SLE and Healthy Individuals

In view of the marked divergence in sex-related AS events in the SLE and healthy subjects, as well as the previously described sex-biased gene expression alterations in whole-blood immune cells [25,26,27,28,29], we examined how genes with sexually DAS compare with those displaying sexually differential gene expression (DGE). First, we identified a total of 111 and 323 genes with sex-dependent DGE in the SLE patients and healthy individuals, respectively (Table 2). These numbers are lower than those of the respective genes with DAS between females and males. Likewise, comparing the SLE and healthy individuals, the number of common genes with sex-biased DGE were fewer than those with sex-biased DAS (25 versus 56, respectively).

**Figure 5:**
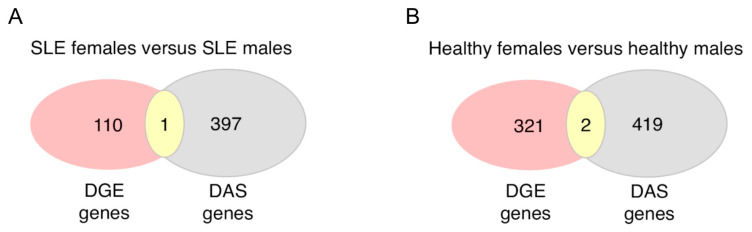
Overlap of genes with sex-dependent differential gene expression and differential alternative splicing in the peripheral blood of active SLE patients (**A**) and healthy individuals (**B**).

Although splicing primarily impacts on mRNA maturation for subsequent translation into proteins, it might also affect the stability of the mRNA molecule, thus influencing the abundance of gene transcripts [7]. To this end, by intersecting genes with sex-biased DAS and sex-biased DGE, we found no overlap except for the *PRKX* gene in the SLE patients and the *MAK* and *HERC5* genes in the healthy subjects (Figure 5). This finding suggests that biological sex may exert distinct effects on gene transcription and RNA splicing with different genes implicated in each process. 

**Figure 6:**
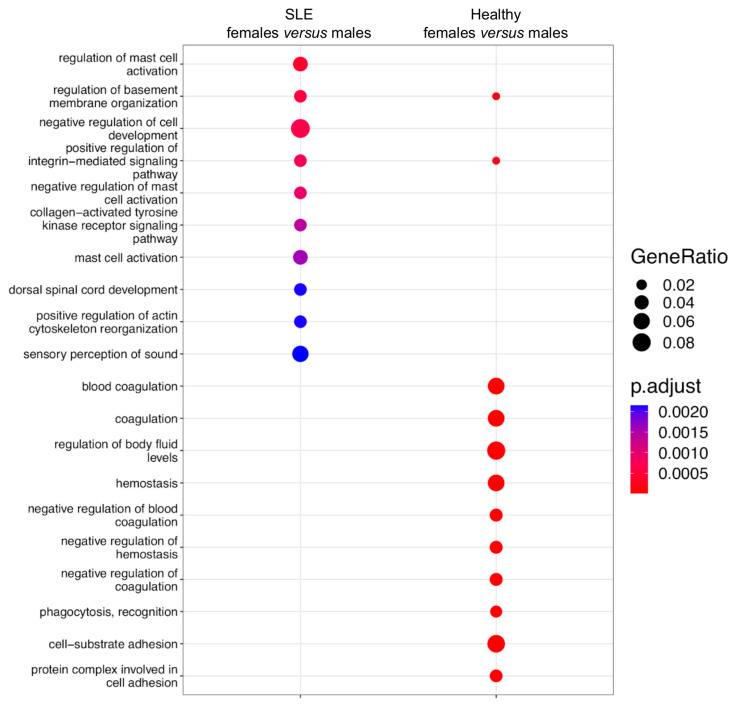
Pathway enrichment analysis of sexually differentially expressed genes in active SLE patients (left column) and healthy subjects (right column).

This result prompted us to investigate whether the molecular processes regulated by sex-related DGE are divergent from those targeted by sex-biased DAS (Figure 2). Our pathway enrichment analysis revealed that sexually differentially expressed genes in SLE were enriched in mast cell activation, cell trafficking/invasion (the regulation of membrane organization) and the integrin-mediated and tyrosine kinase signaling pathways (Figure 6). The respective genes in healthy subjects showed enrichment in coagulation/hemostasis, phagocytosis and cell adhesion. Together, the genomic effect of sex is more prominent at the level of RNA splicing than it is in gene expression, and the integration of these two modalities may provide a more comprehensive view of sex differences in biological functions.

### 3.4. Sex-Biased Intron Retention Events Show Negative Correlation with Corresponding Gene Expression in SLE but Not in Healthy Individuals

**Figure 7:**
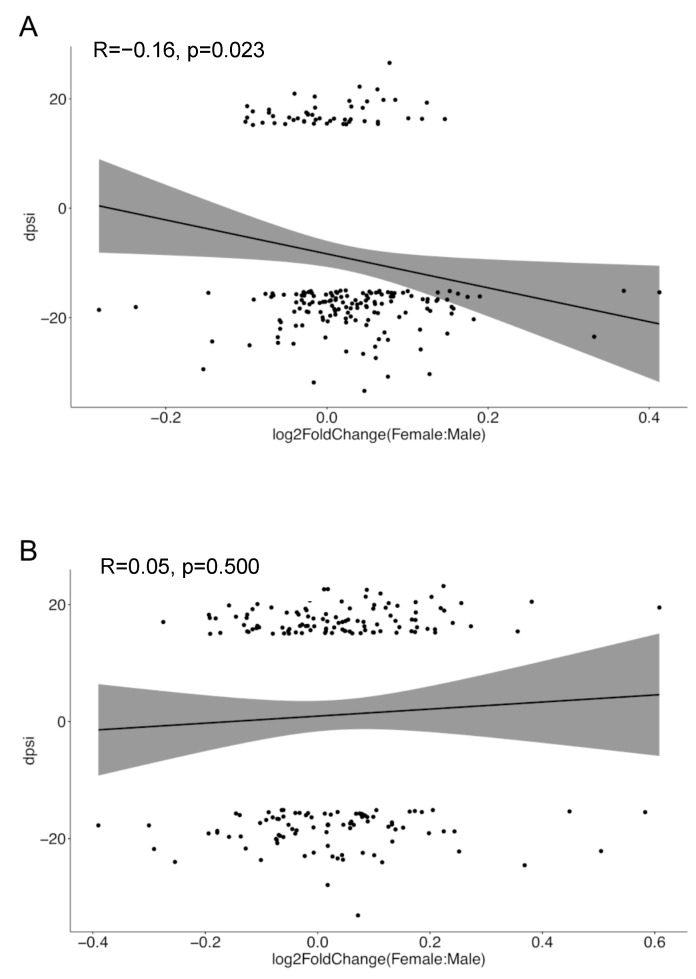
Correlation analysis between sex-related intron-retained genes’ delta-PSI scores and expression levels in SLE (**A**) and healthy (**B**) individuals. The x-axis denotes the log2-fold change in gene expression between females and males, while the y-axis represents the disparity in the intron retention PSI values for this gene. The shaded areas around the regression line represent the 95% confidence intervals for the regression line.

Finally, we shifted our attention to the reduced intron retention events observed in women versus men with SLE as compared to their healthy counterparts (Figure 1). Intron retention has been linked to nonsense-mediated mRNA decay (NMD), thus potentially downregulating gene expression levels, yet these effects are context- and gene-specific [44,45]. We addressed whether sex differences in intron retention were reflected in the respective trends regarding sexual DGE. For this, we focused on the sexually differentially spliced genes harboring intron retention events, and for each gene, we calculated the female-minus-male intron retention-specific delta-PSI value. We then plotted these delta-PSI values against the log2-fold change (female-to-male) in the expression levels of the corresponding genes. This analysis demonstrated an inverse relationship between sex-related intron retention and gene expression in SLE patients (R = −0.16, *p* = 0.023) but not in healthy individuals (Figure 7). Considering that genes with sex-biased DAS and DGE are non-identical (Figure 5), reduced intron retention in SLE females might represent a compensatory mechanism for maintaining appropriate levels of gene expression in the disease inflammatory setting. 

## 4. Discussion

Advances in RNA sequencing approaches, coupled with the emergence of computational capacity, have empowered in-depth research on alternative splicing across the entire transcriptome. Data accumulation and novel methodologies enable researchers to analyze existing data in radically different aspects, as we have done in this study. We performed a comprehensive investigation of the sex-related aspects of AS in whole blood from patients suffering from SLE and healthy individuals. To our knowledge, this is the first detailed analysis of the quantitative and qualitative characteristics of splicing events and underlying genes and pathways according to sex and the presence or absence of autoimmunity (lupus). 

We mapped substantial differences in RNA splicing patterns between the two sexes under both normal conditions and in the context of SLE disease. In both cases, intron retention was the most prevalent class of AS event, followed by exon skipping. Alternative acceptor and alternative donor splicing events occurred only in men, irrespective of the disease state, which suggests that males may be more vulnerable to alternative splicing than females. The basis of such differences remains elusive, although steroid hormones are known to regulate RNA splicing [46]. Certain splicing regulators such as the RNA-binding protein Mbnl3 in eutherian mammals [47] are X-linked, but the question of whether X-chromosome inactivation contributes to human sex differences in AS has not been unexplored. Moreover, the analysis of expression quantitative trait loci (eQTLs) has revealed that sex modifies the effects of functional genetic variants, but it is unclear whether similar effects also pertain to RNA splicing [48]. 

A notable dissimilarity between SLE and healthy sex-dependent AS was the distinct molecular pathways in which the affected genes participate. This is suggestive of the lupus inflammatory milieu altering the AS regulome in a sex-specific manner. In this regard, a previous study on breast cancer reported that IFNγ, through its downstream effector IRF1, is capable of inducing the global AS perturbation of genes involved in the regulation of growth and differentiation, as well as cytokine genes [49]. Similar effects have been described for TNFa [50], the combination of IL-1β and IFNγ [51], and T-cell costimulation through CD28 [52]. Notably, influenza A virus infection is associated with extensive changes in the alternative splicing of host genes [53], but whether or not type I interferon, which is an antiviral cytokine with excessive bioavailability in SLE, can induce such changes has not been demonstrated. Together, these data are in line with the concept that AS represents a dynamic process utilized by cells in response to various endogenous or exogenous stresses. 

Chromatin remodeling and transcription regulation were the main pathways affected by sex-biased AS in SLE. The crosstalk between these processes is well-established, with histone modifications and chromatin configuration creating a platform for splicing factor recruitment and the induction of alternative splicing [54,55]. Thus, the SWI/SNF (mating-type switch/sucrose nonfermenting) complex has been shown to interact with RNA-binding proteins implicated in AS [56,57]. Among the targeted genes in SLE was histone demethylase *KDM6A*, which enables chromatin regions to be exposed for transcription. Supporting our findings, a recent study in human cell lines demonstrated that *KDM6A* mRNAs undergo extensive AS that controls its localization and interaction with other chromatin regulatory complexes [58]. Interestingly, *KDM6A* is an X-linked gene that escapes X-chromosome inactivation in females, and *KDM6A* deletion in CD4+ T-helper cells resulted in autoimmunity amelioration in the context of multiple sclerosis, which is also a female-biased autoimmune disease [59]. 

Another interesting observation is that sex-dependent genomic variation was more pronounced at the level of AS than at the level of gene expression. Notably, the relationship between the extent of splicing perturbations and the cellular function is complex and context-dependent [7]. While an increased number of AS events can be associated with cellular dysfunction, even perturbations in a few genes’ splicing can contribute to disease pathogenesis, as in the case of neurodegenerative disorders [9]. Pending further confirmation and experimental exploitation, our data underscore that RNA splicing may represent an often overlooked source of molecular and functional variability in physiological and pathological settings. The latter has been specifically highlighted in cancer biology, where deregulated AS may contribute to tumorigenesis, immune system evasion and variable response to chemotherapy [60]. The lack of overlap between sexual differentially spliced genes and differentially expressed genes is not unexpected, since AS regulates gene expression, post-transcriptionally producing a remarkable expansion of the genome-coding capacities [61]. The identified alterations in RNA splicing could potentially result in aberrant or non-functional protein isoforms, which can disrupt normal cellular processes or serve as putative autoantigens. Nonetheless, the fact that differential AS occurs, in particular, in genes and functions linked to transcriptional processes such as the uptake and metabolism of nucleobases suggests that this may be reflective of a generalized perturbation under autoimmune/inflammatory conditions, rather than having a causative role. 

To gain insight into sex-dependent genome regulation specifically in the lupus environment, we turned our focus towards genes with sex-dependent AS events in SLE but not in healthy individuals. Some of these genes merit discussion for their potential relevance to autoimmunity. Thus, *PARP14* has previously been implicated in macrophage activation via STAT1 ADP-ribosylation [62]. Another identified gene, *USP19*, negatively regulates type I interferon signaling and positively regulates the autophagy process, which are major immune cell functions previously demonstrated to be deregulated in SLE immune cells [63,64,65]. *RUNX1* is another SLE-specific, sex-dependent AS gene that encodes for a transcription factor involved in major functions of hematopoietic cells, such as hematopoiesis and myeloid differentiation [66]. Importantly, *RUNX1* has been associated with several autoimmune disorders, including SLE [42]. Additionally, one of the genes with the highest degree of sex-biased AS in SLE, compared to the healthy state, was *FDXR* (encoding Ferredoxin Reductase), which is one of the *XIST* RNA interactome genes and displays abnormal expression in activated B cells derived from SLE patients [67]. Collectively, these data serve as a valuable resource for further mechanistic studies and the development of targeted, sex-based diagnostic or prognostic biomarkers in SLE. According to this notion, AS profiles have been proposed as biological predictors for cancer patient survival, with a better accuracy compared to gene expression levels [68,69].

A notable observation was the dissimilarity in intron retention events driven by a reduction in SLE females as compared to SLE males, whereas the opposite trend was observed in the healthy state. Although intron retention has long been neglected and considered as a form of transcriptional ‘noise’, emerging data suggest that it might represent a post-transcriptional gene regulatory mechanism utilized by cells during cell cycle differentiation and the response to stress [45]. We observed a small—yet significant—inverse association between intron-retention-affected genes and their corresponding mRNA expression, specifically in SLE disease. Intron retention abnormalities have previously been described in active-SLE-patient-derived blood immune cells and is associated with the disrupted expression of the underlying genes [20,21]. This is also in line with the study of Ni et al. [70], where most of the genes upregulated in CD4+ T-cells during activation had a reduction in intron retention due to enhanced mRNA stability [70]. The relevance of our results in the context of SLE and sex bias remains to be determined, but nonetheless, it could represent a mechanism for keeping the expression levels of lupus-inducible genes in check. Accordingly, the female/male difference in intron retention in SLE might have accounted for the lack of overlap with sex-biased DGE genes. Nonetheless, one cannot rule out other possible effects of intron retention such as the generation of neoantigens, as reported for malignancies [22,23,24]. 

Given the exploratory nature of our research, it is important to recognize its limitations in reaching definitive conclusions. Although our study provides insights into sex-dependent AS in SLE and healthy individuals, additional confirmatory studies employing methods such as RT-PCR, as well as mechanistic experiments, will be needed to strengthen our observations and establish a solid foundation for future research in this area.

## 5. Conclusions

Our study sheds light on sex-biased AS as a novel, understudied mechanism of genome regulation, both in the context of physiological state and in a female-biased autoimmune disease (SLE). The molecular networks regulated by genes with affected AS versus expression are distinct; therefore, we propose that the simultaneous analysis of gene expression and splicing patterns may provide a more comprehensive view of the processes underlying sex-related diseases. The biological relevance of these findings will require validation in additional patient cohorts coupled with focused mechanistic studies.

## Figures and Tables

**Table 1:** Subgroups of merged RNA sequencing samples used for alternative splicing analysis.

Main Group	Number of Patient Samples	Number of Subgroups
Healthy female	48	5
Healthy male	10	1
SLE female	69	7
SLE male	10	1

**Table 2:** Number of genes with significant sex-dependent differential alternative splicing (DAS) and differential gene expression (DGE) in the whole blood of SLE patients and healthy individuals.

No. Genes	SLE Individuals	Healthy Individuals	Common
Sex-biased differential alternative splicing (DAS)	398	421	56
Sex-biased differential gene expression (DGE)	111	323	25

## Data Availability

All data are available upon reasonable request.

## Supplementary Materials

The following supporting information can be downloaded at: https://www.mdpi.com/article/10.3390/cells12232678/s1, Table S1: Demographic and clinical characteristics in SLE patients and healthy individuals; Table S2: Alternative splicing events demonstrating significant association with disease activity (SLEDAI-2000) in SLE patients; Table S3: Alternative splicing events demonstrating significant association with active renal disease in SLE patients; Table S4: Alternative splicing events demonstrating significant association with serological activity (hypocomplementemia and/or increased anti-dsDNA titers) in SLE patients; Table S5: Sexually differential splicing events in SLE patients; Table S6: Sexually differential splicing events in healthy individuals; Table S7: Shared genes with sexually differential splicing events in SLE and healthy individuals; Table S8: Genes with sexually differential splicing events in SLE but not in healthy individuals.
