# Supplementary material for: Extensive Alternative Splicing Patterns in Systemic Lupus Erythematosus Highlight Sexual Differences
Source: Cells. 2023 Nov 22;12(23):2678. doi: 10.3390/cells12232678 (PMC10705143; doi:10.3390/cells12232678)
Supplement: Supplementary file 1 [file cells-12-02678-s001.zip › Supplementary Table S1.pdf]

Supplementary Table S1. Demographic and clinical characteristics of active SLE patients and healthy individuals analyzed in the present study

	**SLE patients (n=79)**	**Healthy individuals (n=58)**
	n (%) or mean (95% confidence interval)	
Gender (females)	69 (87.3%)	48 (82.8%)
Age (years)	39.9 (36.6–43.2)	40.8 (37.4–44.2)
Race (White)	78 (98.7%)	49 (100%)
No. ACR 1997 criteria	5.21 (4.90–5.53)	–
SLEDAI-2K	8.72 (7.75–9.69)	–
